# Studies on the Ageing of Cement Stabilized Rammed Earth Material in Different Exposure Conditions

**DOI:** 10.3390/ma15031090

**Published:** 2022-01-30

**Authors:** Łukasz Rosicki, Piotr Narloch

**Affiliations:** Faculty of Civil Engineering, Warsaw University of Technology, Al. Armii Ludowej 16, 00-637 Warsaw, Poland; lukasz.rosicki.dokt@pw.edu.pl

**Keywords:** rammed earth, cement stabilized rammed earth, durability, compressive strength, development, seasoning conditions, natural weather conditions

## Abstract

This paper aims to test the deterioration of cement stabilized rammed earth and consider its characteristics during its lifespan in various exposure conditions. Both visual and mechanical properties were tested to determine the impact of long-term exposure to natural weather conditions. Cemented stabilized rammed earth is a variation of the traditional rammed earth building material which has been used since ancient history and is strengthened by the addition of stabilizers in the form of Portland cement. This article analyzes the long-term properties of wall panels made of this material, which were subjected to varying exposure conditions for five years. After this period, compression tests of specimens cut from panels stored in various environmental conditions were carried out. Based on the results and visual properties of the specimens, long-term changes in unconfined compressive strength were observed and primary durability attributes were described. Despite minimal visible wear to the external layers of the wall panels, the natural weather conditions proved to deteriorate material strength characteristics, especially on specimens with high cement content. No correlation between visual characteristics and compressive strength measures were found. The present study is vital in adequately describing cement stabilized rammed earth behavior in natural weather conditions typical of a humid continental climate.

## 1. Introduction

One of the main problems with modern attempts to decrease the anthropogenic environmental impact of buildings is the high level of uncertainty in forecasting the long-lasting properties of newly introduced solutions. Even though rammed earth used to be one of the most popular construction materials used worldwide, the solution properties of modern cement stabilized rammed earth (CSRE) still require further studies, especially focusing on its durability and property alteration through time while being subjected to natural conditions.

Researchers emphasize the need for a broad approach to CSRE assessment [1] as unconfined compressive strength testing in a standardized environment is not enough to provide sufficient information about individual mixtures and specimens, especially considering the material’s possible practical application. Long-term studies [2] focus primarily on erosion measurements, not compressive strength changes which could be decisive while designing buildings with rammed earth-based structures. Such studies usually investigate earth-based composites with no or only trace amounts of an additional binder. In order to find the most sustainable solutions for construction works in the future, precise data considering the characteristics of various materials and their change over time is needed. It is vital to highlight the importance of the local climate condition’s impact on the possible usage of all bio-based, sustainable material. Various countries can be characterized by entirely different needs. Rammed earth applications are traditionally restricted to places with an arid or desert climate with minimal humidity. Most studies from zones with high humidity and harsh winters are usually focused on cement stabilized with agro-industrial wastes [3,4,5].

Traditionally rammed earth walls are constructed with no additional binder, except for clay, by ramming 8–15 cm layers of earthen mixture with no organic components into formwork at optimal water content [6]. These unstabilized materials seem to be outperformed by stabilized solutions which can achieve higher levels of durability and compressive strength.

The compressive strength of both unstabilized and cement stabilized rammed earth has been tested in various past studies (Table 1) [7,8,9,10,11,12,13,14,15,16,17,18], usually after the material has been seasoned for 28 days concurrent with the concrete destructive tests proposed in different standards [19,20,21]. Rammed earth consists of numerous minerals which deteriorate the velocity of development of compressive strength in concrete, primarily due to slowing down the process of hydration. Therefore, it seems valid to assume that the compressive strength of CSRE is still developing in a meaningful way after 28 days. In order to test this statement, measuring destructive tests should be conducted after long lapses of time. Moreover, it seems impractical to season specimens with the required standards as it is impossible to imagine construction works performed over such long periods of time, therefore, in this study, specimen walls were seasoned for five years in normal conditions in addition to the period of standardized seasoning.

The subject presented in this paper is strongly connected with the durability of rammed earth as the influence of climate conditions can be decisive in the usage of these types of materials in various geographical locations. Elements with no protective layers are exposed to moisture and frost which deteriorates their mechanical and visual properties [22]. Rammed earth is characterized by a low wet/dry compressive strength ratio measured between 0.46 and 0.64, even with a 6% addition of cement [9]. Similar results were shown for rammed bricks with a cement content equal to 7.5% [23] with results spanning from 0.47 to 0.52. Similar correlations were shown in wet/dry tests of shear strength conducted on cylinder specimens. Decreases in measured strength for wet specimens were close to 50% [24]. This effect can probably be significantly reduced by the incorporation of limestone residues from the processing of marble, as accelerated degradation caused by successive wetting and drying increased the compressive strength of the specimens [25]. Furthermore, the moisture and kinetic energy of raindrops can decrease the overall quality of rammed earth buildings and elements. It is vital to consider rain-driven erosion, which is the most destructive form of erosion, as CSRE surfaces are subjected to water dropping at 15 to 30° [26].

CSRE, similar to all bio-based materials, has high porosity which can lead to accelerated biodegradation, especially in humid or aqueous conditions. One of the biggest threats is a significant creep, but limited efforts have been devoted to CSRE deformation under constant stress. Nevertheless, constitutive models for primary and steady creep in bio-based concrete were found [27].

Immunity to frost in certain climate zones can be the decisive factor in providing a high level of safety in wide applications of stabilized rammed earth as a construction material. Freeze/thaw testing is one of the methods used to estimate long-term degradation over the accelerated timeframe [26,28,29], but past studies vary greatly in terms of methods and requirements as they are usually governed by national standards for concrete testing and are therefore influenced by varying climate conditions.

Some of these problems can be solved by the utilization of typical thermal and water insulation finishing layers which can dramatically change the thermal response of rammed earth, but can also decrease the sustainability of the whole construction [30,31]. Theoretical predictions [32] show that utilizing CSRE technology guarantees to reduce the embodied energy in typical structures in comparison to traditional burnt clay brick or reinforced concrete. Additionally, non-covered rammed earth walls, if proper architecture solutions are used, can provide stable and adequate levels of temperatures with a very low heating load [33]; this has been proved by in-situ measurements. In the present article the state of current knowledge about rammed earth materials and their properties was developed by and compared with new original long-term studies focused on UCS development in natural outdoor conditions.

There is also a problem with assessing the properties of existing rammed earth construction [34]. which has led to studies considering non-destructive tests [35,36]. Previous research based on artificial intelligence tools [37] proved that the most influential components of CSRE in accordance with UCS are cement and water content followed by clay and silt. The methodologies of most previous studies suggest that destructive tests should be carried out after 28 days of seasoning (similar to most standards for concrete testing) usually in humid conditions with a relative humidity of 50% or more. Few researchers have indicated other points in time, attaining desired moisture as the moment of reaching the measurable compressive strength of the specimens.

There are two main approaches to forming specimens with CSRE. Some researchers chose to inspissate their specimens manually, whereas others chose to use mechanical devices such as a Proctor machine, jackhammer, or hydraulic press. In addition, often there are differences in the number of layers in individual specimens or there may be a complete lack of layers. As shown in Table 1, there is a link between the way the specimens are formed and their density which is highly connected with the compressive strength of composite materials. Additional stabilizers were used, most often in the form of cement or lime. Cement content varied from 3 to 12%. Lime was rarely used solely, it was usually used as an addition to cement in the amount of 1–4% of the mass of the mixture.

Various types of shapes of the specimens were used in compressive strength tests. Cubes and cylinders were used most often, again in similarity with concrete testing, but few researchers decided to conduct their studies on wall panels, prisms, or beams. In the case of CSRE, the shape of the test specimen seems to be of particular importance due to the layered structure of the erected building elements.

Various studies focus on traits other than compressive strength. A clear correlation between fracture energy and tensile strength was proved [38]. The cohesion and friction between layers of CSRE walls were tested, revealing a high level of dependence between the moisture of specimens and their in-plane shear loads [39], and increases in the flexural tensile strength were provided by the addition of gravel fraction [40]. Few studies have tested the corrosion protection of steel embedded in CSRE [41] and the bond strength of rebars in CSRE [42,43], testing the possibility of using CSRE in a similar way as modern reinforced concrete in construction. Results reveal that the correlation between corrosion potential and moisture content within specimens allowed rebar to be depassivated during the designed lifespan. Bond strength is also significantly lower than in modern concrete elements.

Taking this into consideration, it seems warranted to draw a conclusion that the lack of worldwide standards [44] has led to the high level of incompatibility between the presented studies. Numerous variables emerging in the lifespan of a single specimen, from the moment of designing the mixture to conductive compressive strength tests cause valid concern about using measured properties in the wider context and inhibit the utilization of CSRE-based materials. There are two types of goals in the conducted research. Combining past studies with new original long-term tests allows for the measurement and assessment of the physical characteristics of CSRE. Firstly, realistic wear due to weather conditions on five year old CSRE wall panels can be assessed and compared with indoor stored ones. Secondly, UCS change after a long installment of time can be measured. Usually 85% of total shrinkage is recorded within the first 28 days of hardening [45], therefore, during normal conditions deterioration of the construction has exterior causes.

## 2. Materials and Methods

### 2.1. Materials

**Figure 1:**
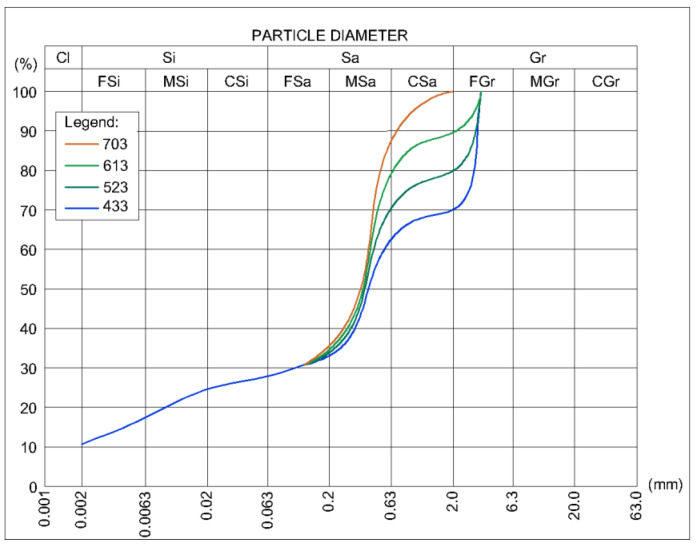
Particle size distribution of soil mixtures used in tests.

A total of eight series of specimens were prepared, differing in grain size and the addition of cement (Table 2). It was decided to apply Portland cement CEM 42.5R as a stabilizer in two different dosages—a minimal amount guaranteeing core durability (6%) and enough UCS for construction applications (9%) allowing us to achieve higher compressive strength results [9,46] and track the differences based on storage condition better. The graining curves of the four soil mixtures from which the specimens were prepared are shown in Figure 1. The three-digit graining symbols represent the proportions of sand, gravel, and silt with clay. For example, the soil mixture symbol 613 means that it is composed of 60% sand, 10% gravel and 30% silty clay. CEM I 42.5 R cement in the amount of 6% or 9% was added to each dry ground mix and mixed dry. Water was added to all mixtures in an amount which guaranteed the optimum moisture content (OMC), i.e., the humidity at which the maximum dry density was obtained by tamping the specimen. The properties of the compacted soil depend on the mineral composition of the soil, especially the content of clay minerals [17,47]. Both soil mixtures contained 30% of silty clay, hence their mineral composition was similar. The soils contained approximately 2.67% of swellable minerals (beidellite). Detailed mineral compositions of the soil mixtures are presented in the article [48].

### 2.2. Methods

#### 2.2.1. Specimen Preparation

**Figure 2:**
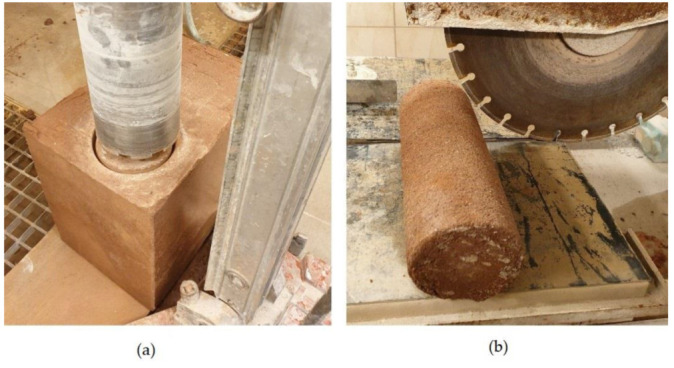
(**a**) Cutting a cylindrical specimen 100 mm in diameter from a rectangular block. (**b**) Trimming the specimen to a height of 100 mm.

The intention of the authors was to prepare the specimens for laboratory tests using a method similar that used when erecting walls from compacted earth on a construction site. For this purpose, a 6.5 kg manual hand rammer was used to compact the load-bearing rammed earth wall. Large specimens of 200 by 300 by 250 mm were compacted in approximately 5 cm layers in a waterproof plywood formwork (Figure 2). The layers were rammed in to the formwork by pulling down the rammer from a height of 30 cm onto the moist layer of soil-cement mixture. The ramming started from one end of the mold, where 10 strokes were made at the very edge, and then moved 5 cm, another 10 strokes were made. When ramming the layers, their volumes were controlled—it was assumed that all the layers of the specimen had to have a similar volume density. After the completion of molding, the specimens were left in forms, tightly wrapped in foil for 24 h, and then demolded. A total of five specimens were prepared for each of the eight series of specimens.

#### 2.2.2. Curing Conditions

Allowing CSRE elements to be subjected to natural weather conditions provides an opportunity to track two opposite effects influencing the mechanical properties of the elements. On the one hand, the compressive strength of the CSRE increases with the progress of over time cement hydration. On the other hand, it may decrease as a result of erosion caused by weather conditions.

Climate context seems to be an important issue when discussing the mechanical properties and durability of construction materials [49,50,51]. Specimens that were stored outdoors were subjected to the conditions shown in Table 3. The testing site was located in Warsaw, Poland, Central Europe. In-situ storage of the specimens lasted from mid-2014 to mid-2019. Warsaw is defined by the Köppen climate classification as Dfb—a humid continental climate, with long cold winters and short summers [52,53]. The urban heat island effect makes winters less severe than in the surrounding nonurbanized areas but provides higher temperature values during long periods of heat, especially by limiting temperature drop during nighttime [53].

As shown in Table 3, during their lifespan the wall panels were submitted to frost for about 240 days and average diurnal temperatures of 10.16 °C with extreme values of 29.3 °C and −15.6 °C. In-situ storage allowed us to subject the specimen to usually omitted factors like wind [average speed of 3.45 km/h] or accelerated surface drying caused by being exposed to natural sunlight. Simultaneously the same number of wall panels were stored indoors, with a constant temperature of 20 °C and a relative humidity of 50%. Specimens were lined up on waterproof foils, with one wall facing south with no obstacle and spaces between the specimens amounting to approximately 10 cm. The specimens were not stored under the roof.

#### 2.2.3. Cutting Specimens for Strength Tests

**Figure 3:**
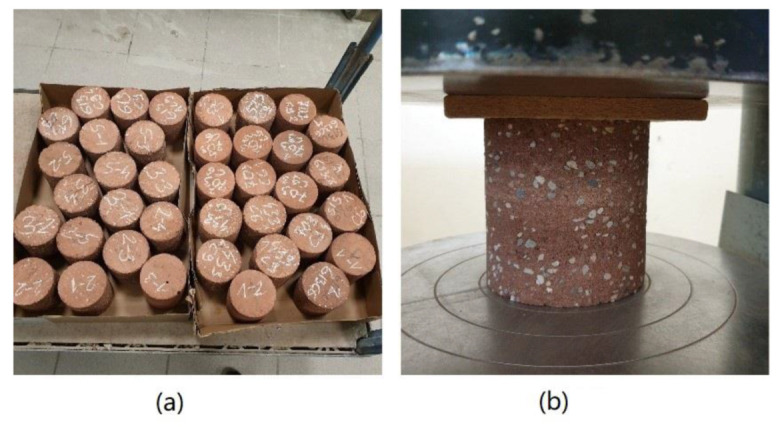
(**a**) Washed cylindrical specimens prepared for compressive strength tests. (**b**) Specimen on a testing machine with soft fiberboard.

A total of four cylindrical specimens 100 mm in diameter and 100 mm were cut from each wall-panel. Two cylindrical forms were cut with a hole saw, then each of the molds was cut to the desired height (Figure 3). Water cutting was not used so as not to change the moisture of the specimens. Specimens were cut and tested on one day.

**Figure 4:**
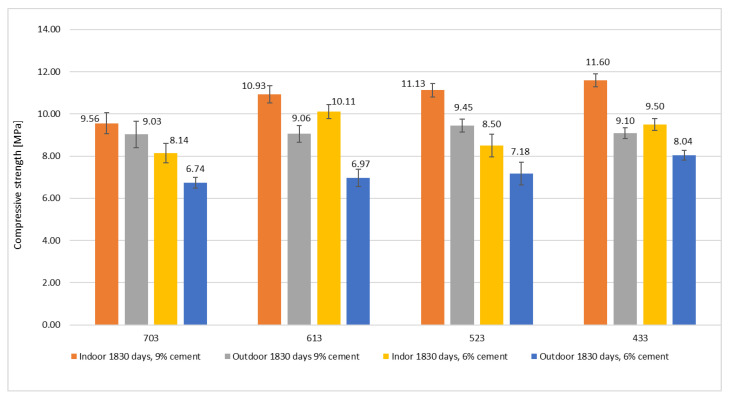
Compressive strength of soil-cement mixtures (mean values with 0.90 confidence interval).

For each of the eight series, at least ten specimens were selected for the compressive strength tests. Since the specimens were not perfectly smooth after cutting, soft fiberboard washers were used between the specimen and the surface of the compressive strength testing machine (Figure 4).

#### 2.2.4. UCS Calculation

The load was continuously applied at a rate of 0.5 MPa/s and failure usually occurred in approximately 20 s. Stress data was recorded in Newtons with a 1% accuracy. Compressive strength for each specimen was calculated as shown in Equation (1).

f_c_ = 4N/πd^2^ (1)

where:

f_c_—compressive strength of single specimen
N—destructive force recorded during test
D—diameter of specimen (arithmetic mean of 3 measurements)

For 10 specimens and a 0.90 decimal confidence level, the two-sided test *p*-value was approximately 1.83. Standard deviation, standards error, and confidence interval values are shown in Table 4.

## 3. Results

All values represent average results from ten or more measurements. Visual ratings of the specimens stored outdoors were conducted on a five level scale. Visible imperfections were noted and assessed. The homogeneity of the specimens’ external layers, amount of loose particles, significant cracks and difference of colors were registered. The homogeneity of the cut core was also taken into consideration. The main reason for using the empirically introduced scale was to compare the mixtures to each other and was not to try to create a universal way of assessing the wear of CSRE elements. Previous studies considering the state of existing rammed earth constructions are usually unique as they often assess one of a kind architectural heritage buildings [54]. Therefore, the type of damages noted in visual inspection can be different and include defects such as biodeterioration, significant erosion and even collapsing [22,55]. Scores are shown in Table 5.

The best scores were achieved by specimens with no gravel added to their mixture. Wall panels with both 6 and 9% cement content were smooth, monochromatic, and homogeneous. Mixture 703-6% has the minimal number of cracks. The worst visual properties were possessed by wall panels with the highest gravel content. The outside layers of these specimens were rugged and uneven with visible layers and discoloration. Applying mixture 433-6% led to a specimen with numerous cracks and loose particles. The differences between the visual aspects of the specimens made out of 613 and 523 were minimal. These mixtures failed to obtain a smooth texture with no discoloration of wall panels. Panels made out of 703 could generally be described as adequate. Correlation between cement content and visual parameters was noted. Specimens with a higher stabilizer content generally presented better visual properties. It is worth noting that after five years these specimens were not significantly different from the ones stored indoors.

The average values of the unconfined compressive strength of outdoor storage specimens varied, based on mixture type and cement content, between 6.74 and 9.45 MPa. UCS differences for 6 and 9% cement mixtures were visible—about 1 to almost 2.5 MPa. The average specimen compressive strength for indoor storage CSRE with 9% cement was higher and achieved 11.6 MPa (Figure 4). Obtained values were significantly lower than those observed in previous similar research [7]. The probable cause is significantly different climate conditions (especially a weak quantity of rainfall), specimen shape, and specimen manufacturing method. As demonstrated, different curing and maturing conditions can cause massive changes in the mechanical properties of rammed earth.

UCS ratios between different types of material are shown in Table 6. Values span from 0.58 to 1.72. For specimens with the same mixture recipe and cement content varying only in curing conditions, differences in compressive strength can mount up to 45%. Changing cement content from 6 to 9% causes a change in UCS up to 34%, while the mixture types themselves can influence UCS only up to 20%. As shown, exposure conditions can be at least as important as mixture type and stabilizer content regarding the final UCS of the material. It is also worth noting that values obtained during research were generally higher than those measured in previous studies based on various standards [19,20,21]. Most researchers fail to obtain values of UCS higher than 6 MPa [8,10,14,17] probably due to a significantly shorter period of compression strength development.

## 4. Discussion

Most previous studies represent highly innovative research that reveal the basic and fundamental principles of testing rammed earth in laboratory conditions and present the wide scope of properties that can be attained by these materials in real life. As shown [16], for example, by the water content of different mixtures, small changes in the dosage of initially introduced water can lead to significant differences in compressive strength. Varying initial conditions and a lack of commonly accepted standards inhibit the utilization of CSRE-based materials on a wider scale. The common application of rammed earth requires in-depth studies on the various physical and mechanical properties of the material on site. Such studies are severely constricted by various climate conditions where such application is possible. This study focuses on material behavior in typical eastern European weather. Previous studies are usually restricted to places with no severe winters where, for example, frost resistance does not affect material applicability [55,56] or is limited to extreme cases [35]. Nevertheless, proper visual and mechanical characteristics of proposed CSRE specimens were maintained after five years of exposure to natural weather conditions.

All UCS results in this study differed due to mixture type, cement content and curing conditions. There is an apparent correlation between the amount of additional stabilizer used and the compressive strength measured. There is also proof of continuous UCS increase after 28 days of maturing as the comparison between five year old indoor stored specimens and 28 day old specimens of mixtures 703, 613, 523,433 with 9% and 6% cement content tested in previous studies showed [17,57]. When taking into consideration the significant deterioration of UCS noted in specimens stored outdoors, it is vital to highlight the fact that the final compressive strength values were higher than corresponding results for specimens tested after 28 days [47,57,58,59].

Long-term outdoor seasoned specimens exhibit lower levels of compressive strength than corresponding materials kept indoors. Overall the condition of the exterior layer can be described as good. Despite not using any protective coverage surfaces, all blocks show minimal visible faults. There were few examples of cracks, loose particles, and discoloration. Despite being influenced by weather conditions for five years, visual properties were kept mostly intact. All surfaces kept similar visual properties to specimens stored indoors despite being subjected to significantly different conditions. The state of the outdoor seasoned panels is almost identical to those stored indoors which may lead to the conclusion that most visual imperfections emerging during the lifespan of specimens is caused by internal factors such as drying and shrinkage. Probably due to inhibited hydration, the results of these processes, such as cracks, are less significant. Mechanical damages during the five year lifespan seem to be negligible for designed mixtures in the addressed climate condition.

A vital part of this study was introducing CSRE specimens to natural conditions and assessing their material behavior and visual attributes after being submitted to natural rainfall and freezing-thawing occurrence. Due to the length of study which was significantly shorter than the average planned duration of any building usage, no final long-term conclusions should be drawn as freeze-thaw deterioration in concrete-based materials can remain undetected after five years especially when specimens are assessed by unaided eyes. No rain-driven erosion was noted as the exterior layers of the wall panels remained relatively smooth. As far as durability is concerned the overall state of all specimens bodes well for possible applications of rammed earth in constructions, but more studies are required. Wall panels made of mixture 433 were characterized by the greatest number of loose particles in the visible layers. Although the 433 mixture achieved the highest possible UCS values, limiting gravel content to 20% seems necessary to ensure proper aesthetic value of uncoated surfaces. As with all on-site experiments, it is essential to emphasize the significance of the weather condition’s’ impact. The tests started mid-year in order to ensure that UCS development began with no freezing cycles. The results are expected to vary if the tests were to begin during winter.

## 5. Conclusions

The present research on five year old specimens, stored both indoors and in natural conditions, allowed us to preliminarily evaluate material properties after long lapses of time. Both the visual and mechanical characteristics of materials were assessed and in many cases proper results were observed.

There was close to no visible deterioration of specimens. Minor defects were noted mainly discoloration, water marks and missing particles. It seems possible to create wall panels that exhibit nonvisible wear even after being subjected to natural conditions for five years with no protective layer. Randomly occurring freeze-thaw cycles and rainfall did not cause visible erosion.

Despite being submitted to natural conditions of a humid continental climate, the specimens preserved their visual properties and kept on increasing in compressive strength.

Obtained values proved that after 28 days of seasoning there can still be significant UCS increase, even when storing rammed earth elements in suboptimal conditions. However, for a few types of mixtures natural weather conditions did cause noticeable decrease of UCS. The results of this research show that exposure conditions can be at least as important as mixture type and stabilizer content regarding the final compressive strength of the wall. There seems to be no correlation between visual characteristic and UCS measurements.

The best mixtures achieved compressive strengths of over 9 MPa even after being subjected to outdoor conditions for years which is similar to values represented by clay bricks that are often used in typical detached houses. Load bearing applications even in humid climate zones with harsh winters seems possible.

It warrants extreme caution while designing constructions with cement stabilized rammed earth as UCS tested after 28 days cannot be representative of the actual properties of the material after longer lapses of time.

Continuous UCS growth is significantly more visible for lower values of cement content which can lead to the conclusion that in such types of mixtures, cement hydration is much more inhibited and attaining desired high values of compressive strength requires longer periods of time than 28 days.

The UCS of cement stabilized rammed earth can be characterized by high volatility caused by various factors. Compressive strength is dependent on agents such as the moisture of the mixture, particle size distribution in dry mixture, stabilizer type and quantity, method of seasoning and age of the specimens. Worldwide standards which allow high precision in property forecasting are yet to be established.

Future studies should focus on finding the precise amount of time after which the compressive strength growth is negligible. Accelerated ageing research on CSRE seems necessary as long-term in-situ studies do not affect the exterior layer of wall panels in a meaningful way.

## Figures and Tables

**Table 1:** The shape of the specimens and the seasoning conditions used in the rammed earth compressive strength tests.

Source	Shape of the Specimen	Stabilizer Type	Compressive Strength [MPa]	Seasoning Conditions
[7]	Cube	Cement 46 MPa, Lime ND ^(1)^	15.40–21.50	Natural exposure, Period length ND ^(1)^
[8]	ND ^(1)^	None	0.90–1.45	Min. 28 days at 20 °C, 75% RH ^(2)^
[9]	Wall-panel	ND ^(1)^	1.82–3.71	Natural exposure, Period length ND ^(1)^
[10]	Block/wall	ND ^(1)^	3.73	28 days at 23 °C, 50% RH ^(2)^
[11]	Cylinder	None	0.25–0.43	27–35 days after attaining equilibrium water content; 20 °C, 57% RH ^(2)^
[12]	Beam	Natural polymer and fibre	2.23–4.44	According to UNE-EN 196-1 2005, UNE-EN 1015-2 and UNE-EN 12190 1998
[13]	Cylinder	None	0.75–2.00	Normal atmosphere, when desired moisture reached wrapped in plastic film for a week
[14]	Prism	Ordinary Portland	3.38–5.44	Cured for 28 days, air dried for two weeks, dried at 50 °C to constant weight in an oven, soaked for 48 h in water, ca. 45 days
[15]	Prism	Ordinary Portland cement of M53 grade	2.00–2.33	Cured under water jug for 28 days
[16]	Beam	None	0.60–7.20	28 days at 23 ± 5 °C, 50 ± 15% RH ^(2)^
[17]	Cube	Portland cement CEM I 42.5 R	1.72–4.99	28 days at 20 °C, 95% RH ^(2)^
[18]	Cylinder	ND ^(1)^	2.52–6.68	1 day in formwork, 7 days in impermeable membrane, ambient condition for next 20 days (maximum mean monthly temperature 18 °C and minimum 7 °C; mean RH 68% ^(2)^)

^(1)^ ND—no data. ^(2)^ RH—relative humidity.

**Table 2:** Specimen series used in the tests.

Specimen Series	Soil Mixture	Cement Addition (%)	Water Content (%) (Equal to OMC)
703-6%	703	6	10
613-6%	613	6	10
523-6%	523	6	9
433-6%	433	6	9
703-9%	703	9	10
613-9%	613	9	10
523-9%	523	9	9
433-9%	433	9	9

**Table 3:** Weather conditions on testing site throughout the years.

Year	Avg. Wind Speed [km/h]	Avg. Temperature [C]	Avg. Relative Humidity [%]	Precipitation [mm]	Number of Days with Avg. Temperature below Zero
2019	3.58	10.93	72.07	390.20	28
2018	3.42	10.51	72.97	235.20	61
2017	3.53	9.45	77.51	339.80	42
2016	3.41	9.84	75.15	277.90	35
2015	3.51	10.39	71.79	172.00	30
2014	3.22	9.87	76.22	251.30	41
Avg.	3.45	10.17	74.29	277.73	39.50

**Table 4:** Weather conditions on testing site throughout the years.

Mixture	Storing Conditions	Cement Content [%]	Standard Deviation	Standard Error	Confidence Intervals
433	Indoor	6	0.483	0.1527	0.280
523	Indoor	6	0.924	0.2922	0.535
613	Indoor	6	0.558	0.1765	0.323
703	Indoor	6	0.803	0.2539	0.465
523	Outdoor	6	0.924	0.2922	0.535
613	Outdoor	6	0.690	0.2182	0.399
433	Outdoor	6	0.386	0.1221	0.223
703	Outdoor	6	0.428	0.1353	0.248
433	Indoor	9	0.523	0.1654	0.303
523	Indoor	9	0.552	0.1746	0.319
613	Indoor	9	0.071	0.0225	0.041
703	Indoor	9	0.853	0.2697	0.494
523	Outdoor	9	0.542	0.1714	0.314
613	Outdoor	9	0.690	0.2182	0.399
433	Outdoor	9	0.441	0.1395	0.255
703	Outdoor	9	1.09	0.3447	0.631

**Table 5:** Results of visual rating.

Mixture Type and Cement Content	Outside Layer Description	Core Description	Visual Rating
703-9%	Smooth, no discoloration, no particle visible, homogenous	Homogenous	5
613-9%	Smooth, minimal graining, discoloration, visible watermarks, visible layers, few particles missing	Few visible particles, slightly uneven edge, visible layers	4
523-9%	Visible layers, minimal discoloration, minimal watermarks, rugged	Visible particles, uneven edge, visible layers	4
433-9%	Rugged, minimal discoloration, visible layers, uneven, graining	Visible particles, loose particles on outside layer, uneven edge	3
703-6%	Smooth, no discoloration, no particle visible, homogenous, few cracks on edges	Homogenous	5
613-6%	Visible layers, minimal discoloration, minimal watermarks, graining, rugged, multiple cracks on edges, numerous particles missing	Few visible particles, slightly uneven edge, visible layers	4
523-6%	Visible layers, minimal discoloration, minimal watermarks, graining, rugged, multiple cracks on edges, numerous particles missing	Visible particles, uneven edge, visible layer	4
433-6%	Rugged, minimal discoloration, visible layers, uneven, visible erosion, multiple cracks on edges, numerous particles missing	Visible particles, loose particles on outside layer, uneven edge	2

**Table 6:** UCS ratio between all material types tested.

-	Mixture			703	613	523	433	703	433	703	613	703	433	703	613	703	433	703	613
Mixture	-	Cement		9%	9%	9%	9%	6%	6%	6%	6%	9%	9%	9%	9%	6%	6%	6%	6%
	Cement	-	Curingcondition	IN	IN	IN	IN	IN	IN	IN	IN	OUT	OUT	OUT	OUT	OUT	OUT	OUT	OUT
		Curing condition	UCS [MPa}	9.56	10.93	11.13	11.60	8.14	10.11	8.50	9.50	9.03	9.06	9.45	9.10	6.74	6.97	7.18	8.04
703	9%	IN	9.56	-	0.87	0.86	0.82	1.17	0.95	1.12	1.01	1.06	1.06	1.01	1.05	1.42	1.37	1.33	1.19
613	9%	IN	10.93	1.14	-	0.98	0.94	1.34	1.08	1.29	1.15	1.21	1.21	1.16	1.20	1.62	1.57	1.52	1.36
523	9%	IN	11.13	1.16	1.02	-	0.96	1.37	1.10	1.31	1.17	1.23	1.23	1.18	1.22	1.65	1.60	1.55	1.38
433	9%	IN	11.6	1.21	1.06	1.04	-	1.43	1.15	1.36	1.22	1.28	1.28	1.23	1.27	1.72	1.66	1.62	1.44
703	6%	IN	8.14	0.85	0.74	0.73	0.70	-	0.81	0.96	0.86	0.90	0.90	0.86	0.89	1.21	1.17	1.13	1.01
433	6%	IN	10.11	1.06	0.92	0.91	0.87	1.24	-	1.19	1.06	1.12	1.12	1.07	1.11	1.50	1.45	1.41	1.26
703	6%	IN	8.5	0.89	0.78	0.76	0.73	1.04	0.84	-	0.89	0.94	0.94	0.90	0.93	1.26	1.22	1.18	1.06
613	6%	IN	9.5	0.99	0.87	0.85	0.82	1.17	0.94	1.12	-	1.05	1.05	1.01	1.04	1.41	1.36	1.32	1.18
703	9%	OUT	9.03	0.94	0.83	0.81	0.78	1.11	0.89	1.06	0.95	-	1.00	0.96	0.99	1.34	1.30	1.26	1.12
433	9%	OUT	9.06	0.95	0.83	0.81	0.78	1.11	0.90	1.07	0.95	1.00	-	0.96	1.00	1.34	1.30	1.26	1.13
703	9%	OUT	9.45	0.99	0.86	0.85	0.81	1.16	0.93	1.11	0.99	1.05	1.04	-	1.04	1.40	1.36	1.32	1.18
613	9%	OUT	9.1	0.95	0.83	0.82	0.78	1.12	0.90	1.07	0.96	1.01	1.00	0.96	-	1.35	1.31	1.27	1.13
703	6%	OUT	6.74	0.71	0.62	0.61	0.58	0.83	0.67	0.79	0.71	0.75	0.74	0.71	0.74	-	0.97	0.94	0.84
433	6%	OUT	6.97	0.73	0.64	0.63	0.60	0.86	0.69	0.82	0.73	0.77	0.77	0.74	0.77	1.03	-	0.97	0.87
703	6%	OUT	7.18	0.75	0.66	0.65	0.62	0.88	0.71	0.84	0.76	0.80	0.79	0.76	0.79	1.07	1.03	-	0.89
613	6%	OUT	8.04	0.84	0.74	0.72	0.69	0.99	0.80	0.95	0.85	0.89	0.89	0.85	0.88	1.19	1.15	1.12	-
			Legend:																
				UCS ratio between different mixtures, same cement content types, same curing condition															
				UCS ratio between same mixtures, different cement content, same curing condition															
				UCS ratio between same mixtures, same cement content, different curing condition															
				Extreme values of UCS ratio															
			IN	Indoor seasoned specimens															
			OUT	Outdoor seasoned specimens															

## Data Availability

More detailed data available on request from authors.

## References

[1] Arrigoni A., Beckett C., Ciancio D., Dotelli G. (2017). Life cycle analysis of environmental impact vs. durability of stabilised rammed earth. Constr. Build. Mater..

[2] Bui Q.B., Morel J.C., Reddy B.V.V., Ghayad W. (2009). Durability of rammed earth walls exposed for 20 years to natural weathering. Build. Environ..

[3] de Azevedo A.R.G., Amin M., Hadzima-Nyarko M., Agwa I.S., Zeyad A.M., Tayeh B.A., Adesina A. (2022). Possibilities for the application of agro-industrial wastes in cementitious materials: A brief review of the Brazilian perspective. Clean. Mater..

[4] Prusty J.K., Patro S.K., Basarkar S.S. (2016). Concrete using agro-waste as fine aggregate for sustainable built environment–A review. Int. J. Sustain. Built Environ..

[5] Ismail Z.Z., Jaeel A.J. (2014). A novel use of undesirable wild giant reed biomass to replace aggregate in concrete. Constr. Build. Mater..

[6] Khan A., Gupta R., Garg M. (2019). Determining material characteristics of “Rammed Earth” using Non-Destructive Test methods for structural design. Structures.

[7] Guettala A., Abibsi A., Houari H. (2006). Durability study of stabilized earth concrete under both laboratory and climatic conditions exposure. Constr. Build. Mater..

[8] Hall M., Damms P., Djerbib Y. (2004). Stabilised rammed earth and the building regulations (2000): Part A-Structural stability. Build. Eng..

[9] Jayasinghe C., Kamaladasa N. (2007). Compressive strength characteristics of cement stabilized rammed earth walls. Constr. Build. Mater..

[10] Miccoli L., Müller U., Fontana P. (2014). Mechanical behaviour of earthen materials: A comparison between earth block masonry, rammed earth and cob. Constr. Build. Mater..

[11] Silva R.A., Oliveira D.V., Miranda T., Cristelo N., Escobar M.C., Soares E. (2013). Rammed earth construction with granitic residual soils: The case study of northern Portugal. Constr. Build. Mater..

[12] Galán-Marín C., Rivera-Gómez C., Petric J. (2010). Clay-based composite stabilized with natural polymer and fibre. Constr. Build. Mater..

[13] Bui Q.B., Morel J.C., Hans S., Walker P. (2014). Effect of moisture content on the mechanical characteristics of rammed earth. Constr. Build. Mater..

[14] Reddy B.V.V., Kumar P.P. (2010). Embodied energy in cement stabilised rammed earth walls. Energy Build..

[15] Raj S., Sharma A.K., Anand K.B. (2018). Performance appraisal of coal ash stabilized rammed earth. J. Build. Eng..

[16] Meimaroglou N., Mouzakis C. (2019). Cation Exchange Capacity (CEC), texture, consistency and organic matter in soil assessment for earth construction: The case of earth mortars. Constr. Build. Mater..

[17] Narloch P.L., Woyciechowski P., Jęda P. (2015). The Influence of Loam Type and Cement Content on the Compressive Strength of Rammed Earth. Arch. Civ. Eng..

[18] Ciancio D., Jaquin P., Walker P. (2013). Advances on the assessment of soil suitability for rammed earth. Constr. Build. Mater..

[19] (2020). Testing of Concrete—Part 4: Strength of Hardened Concrete.

[20] (2019). Building Code Requirements for Structural Concrete and Commentary.

[21] (2003). Specification for Concrete Production.

[22] Luo Y., Zhou P., Ni P., Peng X., Ye J. (2021). Degradation of rammed earth under soluble salts attack and drying-wetting cycles: The case of Fujian Tulou, China. Appl. Clay Sci..

[23] Heathcote K.A. (1995). Durability of earthwall buildings. Constr. Build. Mater..

[24] Lepakshi R., Reddy B.V.V. (2020). Shear strength parameters and Mohr-Coulomb failure envelopes for cement stabilised rammed earth. Constr. Build. Mater..

[25] França B.R., Azevedo A.R.G., Monteiro S.N., Da Costa F., Filho G., Marvila M.T., Alexandre J., Zanelato E.B. (2018). Durability of soil-Cement blocks with the incorporation of limestone residues from the processing of marble. Mater. Res..

[26] Luo Y., Yang M., Ni P., Peng X., Yuan X. (2020). Degradation of rammed earth under wind-driven rain: The case of Fujian Tulou, China. Constr. Build. Mater..

[27] Wu F., Yu Q., Liu C. (2022). Creep characteristics and constitutive model of bio-based concrete in aqueous environment. Constr. Build. Mater..

[28] Leon P., Woyciechowski P., Rosicki Ł., Cichocki D., Lądowej W.I., Warszawska P. (2015). Ziemia ubijana stabilizowana cementem jako materiał konstrukcyjny–ocena nasiąkliwości. Przegląd Bud..

[29] Traoré L.B., Ouellet-Plamondon C., Fabbri A., McGregor F., Rojat F. (2021). Experimental assessment of freezing-thawing resistance of rammed earth buildings. Constr. Build. Mater..

[30] Serrano S., de Gracia A., Cabeza L.F. (2016). Adaptation of rammed earth to modern construction systems: Comparative study of thermal behavior under summer conditions. Appl. Energy.

[31] Narloch P., Protchenko K., Cichocki D. (2019). Hydro-thermal Analysis of Building Envelope Walls with Cement- Stabilized Rammed Earth Structural Layer and Different Thermal Insulators and Their Positioning in Humid Continental Climate Hydro-thermal Analysis of Building Envelope Walls with Cement-Stabi. IOP Conf. Ser. Mater. Sci. Eng..

[32] Reddy B.V.V., Leuzinger G., Sreeram V.S. (2014). Low embodied energy cement stabilised rammed earth building-A case study. Energy Build..

[33] Soudani L., Woloszyn M., Fabbri A., Morel J.C., Grillet A.C. (2017). Energy evaluation of rammed earth walls using long term in-situ measurements. Sol. Energy.

[34] Rodríguez-Mariscal J.D., Canivell J., Solís M. (2021). Evaluating the performance of sonic and ultrasonic tests for the inspection of rammed earth constructions. Constr. Build. Mater..

[35] Beckett C.T.S., Jaquin P.A., Morel J.C. (2020). Weathering the storm: A framework to assess the resistance of earthen structures to water damage. Constr. Build. Mater..

[36] Canivell J., Martin-del-Rio J.J., Alejandre F.J., García-Heras J., Jimenez-Aguilar A. (2018). Considerations on the physical and mechanical properties of lime-stabilized rammed earth walls and their evaluation by ultrasonic pulse velocity testing. Constr. Build. Mater..

[37] Anysz H., Brzozowski Ł., Kretowicz W., Narloch P. (2020). Feature importance of stabilised rammed earth components affecting the compressive strength calculated with explainable artificial intelligence tools. Materials.

[38] Arto I., Gallego R., Cifuentes H., Puertas E., Gutiérrez-Carrillo M.L. (2021). Fracture behavior of rammed earth in historic buildings. Constr. Build. Mater..

[39] Pavan G.S., Ullas S.N., Rao K.S.N. (2020). Interfacial behavior of cement stabilized rammed earth: Experimental and numerical study. Constr. Build. Mater..

[40] Narloch P.L., Lidner M., Kunicka E., Bielecki M. (2015). Flexural tensile strength of construction elements made out of cement stabilized rammed earth. Procedia Eng..

[41] Meek A.H., Beckett C.T.S., Carsana M., Ciancio D. (2018). Corrosion protection of steel embedded in cement-stabilised rammed earth. Constr. Build. Mater..

[42] Lepakshi R., Reddy B.V.V. (2020). Bond strength of rebars in cement stabilised rammed earth. Constr. Build. Mater..

[43] Lepakshi R., Reddy B.V.V., Reddy B.V.V., Mani M., Walker P. (2019). Bond Strength of Rebars in Cement-Stabilised Rammed Earth BT Earthen Dwellings and Structures: Current Status in Their Adoption.

[44] Kaliszuk-Wietecka A., Leon P. (2014). Konstrukcyjne zastosowanie surowej ziemi jako materiału budowlanego. Przegląd Bud..

[45] Woyciechowski P., Narloch P.L., Cichocki D. (2017). Shrinkage characteristics of cement stabilized rammed earth. MATEC Web Conf..

[46] Kariyawasam K.K.G.K.D., Jayasinghe C. (2016). Cement stabilized rammed earth as a sustainable construction material. Constr. Build. Mater..

[47] Narloch P., Woyciechowski P., Kotowski J., Gawriuczenkow I., Wójcik E. (2020). The effect of soil mineral composition on the compressive strength of cement stabilized rammed earth. Materials.

[48] Rogala W., Anysz H., Narloch P. (2021). Designing the Composition of Cement-Stabilized Rammed Earth with the Association Analysis Application. Materials.

[49] Ávila F., Puertas E., Gallego R. (2020). Characterization of the mechanical and physical properties of unstabilized rammed earth: A review. Constr. Build. Mater..

[50] Saeid G., Vahab T. (2020). Durability of Rammed Earth Materials. Int. J. Geomech..

[51] Narloch P.L., Woyciechowski P., Dmowska E., Halemba K. (2015). Durability Assessment of Monolithic Rammed Earth Walls. Arch. Civ. Eng..

[52] Peel M.C., Finlayson B.L., McMahon T.A. (2007). Updated world map of the Köppen-Geiger climate classification. Hydrol. Earth Syst. Sci..

[53] Błażejczyk K., Baranowski J., Jendritzky G., Błażejczyk A., Bröde P., Fiala D. (2015). Regional features of the bioclimate of central and southern europe against the background of the köppen-geiger climate classification. Geogr. Pol..

[54] Luo Y., Zhong H., Bao F., Guo Z., Ni P. (2022). Insights into natural and carbonation curing of ancient Chinese rammed earth mixed with brown sugar. Constr. Build. Mater..

[55] Martín-del-Rio J.J., Canivell J., Torres-González M., Mascort-Albea E.J., Romero-Hernández R., Alducin-Ochoa J.M., Alejandre-Sánchez F.J. (2021). Analysis of the materials and state of conservation of the medieval rammed earth walls of Seville (Spain). J. Build. Eng..

[56] Cid-Falceto J., Mazarrón F.R., Cañas I. (2012). Assessment of compressed earth blocks made in Spain: International durability tests. Constr. Build. Mater..

[57] Anysz H., Narloch P. (2019). Designing the composition of cement stabilized rammed earth using artificial neural networks. Materials.

[58] Narloch P., Hassanat A., Tarawneh A.S., Anysz H., Kotowski J., Almohammadi K. (2019). Predicting compressive strength of cement-stabilized rammed earth based on SEM images using computer vision and deep learning. Appl. Sci..

[59] Narloch P., Woyciechowski P. (2020). Assessing Cement Stabilized Rammed Earth Durability in A Humid Continental Climate. Buildings.

